# What explains the inequalities in health care utilization between immigrants and non-migrants in Switzerland?

**DOI:** 10.1186/s12889-021-10393-9

**Published:** 2021-03-18

**Authors:** Christina Tzogiou, Stefan Boes, Beatrice Brunner

**Affiliations:** 1 Winterthur Institute of Health Economics, Zurich University of Applied Sciences (ZHAW), Gertrudstrasse 15, Winterthur, 8401 Switzerland; 2 Department of Health Sciences and Medicine, University of Lucerne, Frohburgstrasse 3, Lucerne, 6002 Switzerland

**Keywords:** Inequalities, Immigrants, Health care demand, Non-linear decomposition

## Abstract

**Background:**

Inequalities in health care use between immigrants and non-migrants are an important issue in many countries, with potentially negative effects on population health and welfare. The aim of this study is to understand the factors that explain these inequalities in Switzerland, a country with one of the highest percentages of foreign-born population.

**Methods:**

Using health survey data, we compare non-migrants to four immigrant groups, differentiating between first- and second-generation immigrants, and culturally different and similar immigrants. To retrieve the relative contribution of each inequality-associated factor, we apply a non-linear decomposition method and categorize the factors into demographic, socio-economic, health insurance and health status factors.

**Results:**

We find that non-migrants are more likely to visit a doctor compared to first-generation and culturally different immigrants and are less likely to visit the emergency department. Inequalities in doctor visits are mainly attributed to the explained component, namely to socio-economic factors (such as occupation and income), while inequalities in emergency visits are mainly attributed to the unexplained component. We also find that despite the universal health care coverage in Switzerland systemic barriers might exist.

**Conclusions:**

Our results indicate that immigrant-specific policies should be developed in order to improve access to care and efficiently manage patients in the health system.

**Supplementary Information:**

The online version contains supplementary material available at (10.1186/s12889-021-10393-9).

## Background

Differences in health care utilization between immigrants and non-migrants are well documented in the literature and are often associated with adverse health and welfare effects [1–6]. But what can explain these differences? Ethnicity and socio-economic status are two likely driving forces behind the observed pattern [1, 6–9]. In particular, cultural differences, lack of information (linked to language), as well as legal and administrative barriers may all contribute to the unequal health care use and access to services. A European study on the elderly shows that health system characteristics (e.g., supply-side factors, health insurance) may at least partly explain differences in health care use between immigrants and non-migrants [10]. Moreover, a lower utilization of care among immigrants may also be explained by the healthy migration effect [7, 11].

Switzerland is a particularly interesting country to study migration-related inequalities. First, approximately 26% of the population are foreign-born nationals [12], and they constitute 26% of the working population [13]. At the same time, immigrants in Switzerland form a heterogeneous population. The Free Movement of Persons Agreement signed with the European Union marked a labor liberalization in Switzerland by triggering a large influx of economic immigrants [14]. This agreement grants equal living, employment and mobility opportunities to any national of an EU-27 or EFTA state with employment in Switzerland. The entrance of refugees or other non-economic immigrants is regulated in Switzerland on the basis of the Federal Asylum Act and the Dublin Agreement and without applying quotas. Quotas only apply to third-country economic immigrants. Second, Switzerland has an advanced health system, with a good provider network and guaranteed access to care through mandatory health insurance [15, 16]. For asylum seekers, the mandatory basic health insurance is covered by the cantons (Federal Act of 18 March 1994 on Health Insurance). However, there is evidence that some immigrant groups tend to suffer higher rates of mortality and morbidity compared to non-migrants [5, 17], which could be due to differences in the utilization of care [4, 18].

The aim of this study is to examine the factors that are associated with the inequalities in health care utilization between different immigrant groups and non-migrants in Switzerland. Inequalities are referred to the observed differences in mean outcomes between the two groups. We conduct a non-linear decomposition analysis based on the well-known Oaxaca-Blinder approach using linked health survey data for the general and the migrant population. We distinguish between four immigrant groups (first- and second-generation, as well as culturally different and similar immigrants) and partition the associated factors into demographic, socio-economic, health insurance and health status factors. The main inequality measures are the extensive margins of doctor and emergency department (ED) visits during the past twelve months, but we also assess inequalities at the intensive margins.

We contribute to the previous literature mainly in four ways. First, while previous studies have evaluated the relative risks of health care utilization for particular groups, they have rarely looked into the explaining factors. Building on a theoretical model for health care use, we identify the factors and the way they are associated with inequalities in health care utilization, and we quantify their relative contribution to these inequalities. To this aim, we apply a non-linear decomposition method, which constitutes our second contribution. This method has rarely been applied to the study of inequalities in health care utilization. By decomposing the means compared to the concentration index, which is mostly analyzed in this field, we do not consider inequalities only in one dimension that follows a natural ordering [3]. In contrast, the method we apply decomposes the inequality in the mean outcome into an explained component, attributed to differences in observed predictors (characteristics), and an unexplained component, attributed to differences in associations of these predictors and the outcome variables (coefficients). The non-linearity of the decomposition method constitutes our third contribution, as this method has been applied mainly for linear regression models. Non-linear analyses, however, performed better than the linear alternative in the goodness-of-fit tests that we performed. Finally, we contribute to the literature by distinguishing between different types of explanatory factors. For policy-makers, modifiable factors are especially relevant. Thus, understanding how these factors are associated with inequalities in health care utilization can be leveraged in the design of policy interventions that aim to reduce inequities in health care utilization and establish a sustainable solidarity-based health care system. In particular, knowledge about these factors can be leveraged to improve access to care, promote equal quality of care, and steer patients efficiently through the health system. This in turn could reduce spending and improve the overall efficiency of the health system. Cost containment is particularly important considering that Switzerland has among the highest health care costs in the world (US$ 788 per capita per month [19]) and out-of-pocket payments (28.9% [19]), making health care less affordable for low-income groups. In addition, equal health conditions are an essential component of integration, thus playing an important role for society.

The remainder of the paper is structured as follows. The next section provides an overview of the data sources and the non-linear decomposition approach used in our study. The results section describes the inequalities in health care utilization in Switzerland and presents the results of the decomposition. The last two sections discuss the results and conclude the paper.

## Methods

### Data

The data for this study are drawn from the Swiss Health Survey (SHS) 2012, provided by the Federal Statistical Office [20], and the Second Health Monitoring of the Migrant Population in Switzerland 2010 (GMM II), provided by the Federal Office of Public Health [21]. The two cross-sectional datasets contain information on health status, health-related behavior, health care utilization, and various socio-economic characteristics.

The SHS is based on a random sample of households in Switzerland and has been conducted every five years since 1992. It represents the permanent resident population aged 15 and above. The data are collected by telephone interviews conducted in German, French and Italian, followed by a written questionnaire. Due to the language restriction, people who do not speak any of the national languages in Switzerland are not included, and thus, the SHS alone is not well suited to investigate immigrant-non-migrant differences in health care utilization.

The GMM is a telephone survey based on the SHS and monitors the health outcomes in a sample of immigrants with particular nationalities. This disproportionate, stratified sample is drawn randomly from the central information system for immigrants in Switzerland (ZEMIS). GMM has been carried out twice so far, in 2004 (GMM I) and 2010 (GMM II). GMM II focuses only on immigrants from the permanent foreign population coming from Portugal, Turkey, Kosovo and Serbia as well as asylum-seeking or asylum-granted immigrants from two countries, namely Sri Lanka (Tamils) and Somalia. For GMM II, the German version of the SHS 2007 was translated into French, Portuguese, Turkish, Albanian, Serbian, Tamil and Somali.

We combine the two datasets in order to obtain a more comprehensive sample of the immigrant population in Switzerland. We chose the SHS 2012, instead of the most recent wave from 2017, as the former is only two instead of seven years apart from the most recent GMM II in 2010. GMM II has been combined before with SHS 2007 [5], but only the Swiss sub-sample was extracted. In contrast, we use the whole sample from the SHS, including all immigrants, and benefit from a smaller time distance between the two surveys.

The immigrant population in the combined sample is not representative for Switzerland because GMM II focuses on particular countries, and the SHS excludes certain immigrant groups. For this reason, we apply a probability weighting approach, which corresponds to the inverse of the probability of being included in the sample by nationality. Weights are calculated as *N*/*n*, where *N* is the number of people in a particular population group according to the Population and Households Statistics provided by the Federal Statistical Office [22], and *n* is the number of people in that group in the sample. We also impose an age restriction on our sample, because GMM II only includes immigrants between 17 and 73 years old.

### Definition of non-migrants and immigrant groups

We consider individuals with Swiss nationality as non-migrants, including foreign-born nationals. This is justified on the basis of the stringent legal requirements for naturalization according to the Federal Act of Swiss Citizenship [23]. In particular, the residence in Switzerland for a minimum duration of ten years, the successful integration, the familiarity with the Swiss way of life and no threat to public security need to be proven. Additionally, the mean residence duration of the foreign-born nationals in our data set is 28 years.

Due to the heterogeneity of the immigrant population in Switzerland, we distinguish between four immigrant groups. *First-generation* immigrants are defined as foreign nationals that were not born in Switzerland. *Second-generation* immigrants are defined as foreign nationals that were born in Switzerland.

We further distinguish immigrants with respect to two country-of-origin-specific value dimensions defined by Inglehart and Baker [24] and classify them to *culturally different* and *culturally similar*. We hypothesized that cultural values are associated with the perception and use of health care services. This hypothesis is supported by the findings of Roudijk et al. [25] who show that cultural values are associated with differences in self-reported health. Similarly, Mackenbach [26], using empirical evidence from 42 European countries, also shows that culture may partly determine differences in health behaviors and health outcomes. Therefore, we expect immigrants with a similar value system to Swiss non-migrants to display similarities in healthcare utilization.

Using data from the Word Value Survey Inglehart and Baker [24] found that human attitude is associated with the values dominating in their country-of-origin and identified two main dimensions of cross-cultural variation in the world. The first dimension reflects traditional versus secular-rational values and the second dimension reflects survival versus self-expression values. Traditional values emphasize the importance of religion, family and obedience, national pride and respect for authority. Secular-rational values are associated with the transition from an agrarian to an industrial society and emphasize values such as tolerance of diversity and self-expression. In societies where survival is not taken for granted, tolerance of ethnic and cultural diversity, subjective well-being and environmental consciousness are low, while materialist values and economic and physical security are emphasized. In societies where survival is taken for granted, these values shift in the opposite direction towards self-expression and tolerance of diversity.

Based on the distribution of the countries along these two value dimensions we followed Brunner and Kuhn [27] and defined immigrants as *culturally similar* if they have a similar value system to Swiss non-migrants, characterized by the relative importance of secular-rational (as opposed to traditional) and self-expression (as opposed to survival) values. This group includes people from European Christian, non ex-communist countries and English-speaking OECD countries (see list of countries in notes of Table 1). The remaining immigrants are defined as *culturally different*, as they differ either on one or on both value dimensions from non-migrants in Switzerland.

**Table 1:** Share of immigrants in the sample by different groups, in %

	overall	1st generation	2nd generation	culturally different	culturally similar
	25	88	12	36	64
Top 1	Germany, 17	Germany, 18	Italy, 38	former Yugoslavia, 45	Germany, 26
Top 2	Italy, 16	former Yugoslavia, 16	former Yugoslavia, 18	Turkey, 11	Italy, 26
Top 3	former Yugoslavia, 16	Italy, 14	Portugal, 14	Sri Lanka, 4	Portugal, 19
Top 4	Portugal, 12	Portugal, 12	Spain, 9	Brazil, 3	France, 9
Top 5	France, 6	France, 6	Turkey, 5	Poland, 3	Spain, 6
1st generation	88			91	87
2nd generation	12			9	13
Number of					
observations	5446	4771	675	2806	2640

Source: SHS 2012, GMM II 2010, own calculations.

Notes: Culturally different countries: Afghanistan, Albania, Algeria, Angola, Argentina, Armenia, Bolivia, Brazil, Bulgaria, Burundi, Cambodia, Cameroon, Cape Verde, Central Africa, Chile, China, Colombia, Congo, Costa Rica, Côte d’Ivoire, Cuba, Czech Republic, Dominican Republic, Ecuador, Egypt, Eritrea, Ethiopia, former Yugoslavia, Gambia, Georgia, Guinea, Haiti, Hungary, India, Indonesia, Iraq, Iran, Israel, Japan, Kenya, Latvia, Lebanon, Lithuania, Madagascar, Mali, Mauritius, Mexico, Moldova, Mongolia, Morocco, Niger, Pakistan, Palestine, Peru, Philippines, Poland, Romania, Russia, Rwanda, Senegal, Slovakia, Somalia, South Africa, Sri Lanka, Thailand, Togo, Tunisia, Turkey, Ukraine, Uzbekistan, Venezuela.

Culturally similar countries: Australia, Austria, Belgium, Canada, Denmark, Finland, France, Germany, Greece, Ireland, Italy, Lichtenstein, Luxembourg, Malta, Netherlands, Portugal, Spain, Sweden, United Kingdom, United States

There are different methods to classify immigrants and there is no consensus across international and Swiss studies. We, therefore, conducted further analyses in the Additional Files classifying immigrants to those 1. from Switzerland’s neighbouring countries, 2. from other European countries and 3. from other non-European countries. Finally, based on the Swiss naturalization requirements we distinguished between immigrants with a residence duration of at least ten years and immigrants with a residence duration smaller than ten years.

### Predictors of health care utilization

According to Andersen’s behavioral model of health services use [28], there are three types of factors that explain health care utilization: first, predisposing factors that include the individual’s demographic characteristics, social structures and health beliefs that determine his or her disposition to use health care services; second, enabling factors that refer to the individual’s personal, family and community resources, which can enable use through lower access costs; third, need factors that indicate the perceived and evaluated health status, which can explain care-seeking and adherence to a medical regime.

Age, gender, marital status, education and occupation are included as predisposing factors. The enabling factors include language region, urban area, income, and level of insurance deductible. As a need indicator, we include activities of daily living (ADL), as it is a more objective measure of need compared to self-assessed health status or chronic illness. All variables are described in Table S1 in the Additional Files. Due to data constraints, we cannot consider other factors that could also be associated with health care utilization, such as family structures or parents’ country of birth. We also cannot consider factors that do not vary in both groups, such as speaking one of the national languages in Switzerland. This factor does not vary in the Swiss group, as this group was extracted from the SHS, which was conducted in all three national languages. As a result, all Swiss speak by definition one of the national languages.

### Decomposition analysis

We apply a non-linear multivariate decomposition method based on logit and negative binomial II regression models following the well-known Oaxaca-Blinder approach [29, 30]. This method allows us to decompose the observed inequalities between immigrants and non-migrants into two components. Equation 1 shows the decomposition of the observed differences in mean outcomes $\bar {Y}$ between Swiss (SUI = comparison group) and each immigrant group (IMM = reference group, defined above):

1 $$ \begin{aligned} \bar{Y}_{SUI} - \bar{Y}_{IMM} =& G\left(X'\hat{\beta}_{SUI}|D_{SUI}=1\right) - G\left(X'\hat{\beta}_{IMM}|D_{IMM}=1\right) \\ =&\underbrace{G\left(X'\hat{\beta}_{IMM}|D_{SUI}=1\right) -G\left(X'\hat{\beta}_{IMM}|D_{IMM}=1\right)}_{\text{explained component}} \\ &+\underbrace{G\left(X'\hat{\beta}_{SUI}|D_{SUI}=1\right) - G\left(X'\hat{\beta}_{IMM}|D_{SUI}=1\right)}_{\text{unexplained component}} \\ \end{aligned}  $$

where *D*_*SUI*_ and *D*_*IMM*_ represent indicators for Swiss non-migrants and immigrants, respectively. *X* is the vector of predictors described in the previous section. *G*(*X*^′^*β*) describes the mean function of the outcome *Y*, which is a logit function in the case of binary outcomes (extensive margin of doctor and ED visits) and an exponential function in the case of count outcomes (intensive margins). The vectors *β* are estimated separately for non-migrants (SUI) and the four immigrant groups (IMM). The first component of Eq. (1) is explained by differences in the observed predictors (characteristics), while the second is the unexplained component, which derives from differences in coefficients.

This decomposition method has so far been applied mainly for linear regression models. Non-linear applications are rare in health economics. We choose non-linear decomposition based on goodness-of-fit analyses^1^. In addition, as Fortin, Lemieux and Firpo [31] have noted, “non-linear decomposition may perform better than the linear alternative (linear probability model, LPM) when the gap is located in the tails of the distribution or when there are very large differences in the explanatory variables, whose effects would remain unbounded in a LPM.” Extensions of the Oaxaca-Blinder decomposition method for non-linear models have been suggested in the literature [32–37]. We apply the non-linear multivariate decomposition method proposed by Powers, Yoshioka and Yun [37] using the *mvdcmp* command in Stata. One of the advantages of this method is that the results are insensitive to the order to which the predictors are inserted into the decomposition (path dependency problem). They also overcome the omitted group [31] or identification [38, 39] problem associated with the choice of reference group when categorical variables are included among the predictors (for more information, see [37]). Another valuable feature of the command is that it allows for a detailed decomposition that retrieves the relative contribution of each predictor along with robust standard errors for the explained and unexplained components.

### Additional analysis

Not only are immigrants a heterogeneous group in Switzerland but also non-migrants, especially with respect to the language region. As a result, inequalities in health care utilization between immigrants and non-migrants could potentially vary across regions. For example, the health care utilization of French immigrants might be more similar to French-speaking non-migrants than to German-speaking non-migrants. The GMM II data allows us to distinguish only between German- and Latin-speaking regions. We therefore conduct an additional sub-sample analysis using only the SHS data, which allows us to differentiate between all three language regions in Switzerland.

## Results

### Sample description

Table 1 shows the distribution of immigrants across the four immigrant groups defined in page 9, along with the top five most frequent foreign nationalities within each group. The combined sample consists of 19991 observations, 25% of which are immigrants. 88% of the immigrants are first-generation and 12% second-generation, 36% are culturally different and 64% culturally similar.

Table 2 presents the main characteristics of our sample for non-migrants and each immigrant group. Compared to non-migrants, all immigrants are on average significantly younger (31 to 44 years old versus 47 years old) and a higher share of the immigrants are male (48% to 57% versus 47%). Compared to non-migrants, also a higher share of immigrants live in urban areas (approximately 82% versus 69%), while a lower share of immigrants live in the German-speaking region, except for culturally different immigrants (55% to 59% versus 68%). Moreover, all but the culturally similar immigrants have a lower equivalized monthly household income, and a higher share of immigrants are unemployed compared to non-migrants. More first-generation and culturally similar immigrants and fewer second-generation immigrants have completed a tertiary education compared to non-migrants. The latter might be attributed to the lower average age. Immigrants more similar to non-migrants (i.e., second-generation and culturally similar immigrants) are less likely to have impaired activities of daily living due to health problems. An explanation could be again that immigrants (especially second generation) are on average younger.

**Table 2:** Descriptive statistics by immigrant groups

	Swiss	Immigrants				
		overall	1st generation	2nd generation	culturally different	culturally similar
Demographic factors						
age	46.6	40.4^***^	41.7^***^	31.1^***^	34.6^***^	43.8^***^
		(13.6)	(13.6)	(10.8)	(11.8)	(13.7)
male	0.469	0.519^***^	0.512^***^	0.573^***^	0.482	0.541^***^
		(0.499)	(0.500)	(0.497)	(0.500)	(0.499)
married	0.571	0.615^***^	0.655^***^	0.308^***^	0.673^***^	0.582
		(0.479)	(0.461)	(0.447)	(0.461)	(0.492)
German-speaking region	0.676	0.593^***^	0.594^***^	0.592^***^	0.673	0.548^***^
		(0.487)	(0.488)	(0.477)	(0.458)	(0.500)
urban	0.687	0.824^***^	0.823^***^	0.826^***^	0.825^***^	0.823^***^
		(0.386)	(0.383)	(0.404)	(0.382)	(0.389)
Socio-economic factors						
compulsory education	0.100	0.262^***^	0.272^***^	0.187^***^	0.304^***^	0.239^***^
		(0.470)	(0.474)	(0.430)	(0.484)	(0.449)
secondary education	0.726	0.475^***^	0.444^***^	0.709	0.503^***^	0.459^***^
		(0.500)	(0.498)	(0.463)	(0.500)	(0.498)
tertiary education	0.174	0.263^***^	0.283^***^	0.104^***^	0.193	0.302^***^
		(0.393)	(0.406)	(0.250)	(0.326)	(0.441)
household income	4607	4250^***^	4276^***^	4049^***^	3355^***^	4758^*^
		(2333)	(2385)	(1926)	(1788)	(2580)
intern	0.025	0.030	0.019^*^	0.109^***^	0.049^***^	0.019^*^
		(0.198)	(0.159)	(0.353)	(0.231)	(0.154)
ordinary employee	0.140	0.221^***^	0.207^***^	0.327^***^	0.297^***^	0.178^***^
		(0.451)	(0.446)	(0.479)	(0.479)	(0.406)
employee with leadership	0.415	0.340^***^	0.342^***^	0.327^***^	0.249^***^	0.392^*^
		(0.443)	(0.444)	(0.435)	(0.367)	(0.486)
director/chief	0.097	0.095	0.101	0.044^***^	0.031^***^	0.131^***^
		(0.250)	(0.258)	(0.182)	(0.145)	(0.319)
self-employed	0.070	0.043^***^	0.043^***^	0.043^*^	0.028^***^	0.051^***^
		(0.189)	(0.192)	(0.170)	(0.162)	(0.214)
inactive	0.237	0.176^***^	0.193^***^	0.050^***^	0.150^***^	0.191^***^
		(0.347)	(0.360)	(0.203)	(0.304)	(0.383)
unemployed	0.016	0.095^***^	0.094^***^	0.100^***^	0.196^***^	0.038^***^
		(0.370)	(0.373)	(0.346)	(0.447)	(0.202)
Health insurance factors						
deductible = 300	0.302	0.239^***^	0.239^***^	0.240^**^	0.261^**^	0.226^***^
		(0.423)	(0.424)	(0.416)	(0.423)	(0.423)
deductible = 500	0.130	0.127	0.128	0.120	0.112	0.136
		(0.335)	(0.338)	(0.313)	(0.316)	(0.353)
deductible = 1000	0.056	0.049	0.049	0.049	0.034^***^	0.058
		(0.212)	(0.210)	(0.222)	(0.190)	(0.232)
deductible ≥1500	0.326	0.268^***^	0.272^***^	0.243^***^	0.184^***^	0.316
		(0.414)	(0.416)	(0.397)	(0.354)	(0.457)
deductible = missing	0.186	0.316^***^	0.312^***^	0.348^***^	0.409^***^	0.264^***^
		(0.483)	(0.482)	(0.494)	(0.499)	(0.443)
Health status factor						
ADL	0.219	0.208	0.214	0.163^**^	0.239	0.191^**^
		(0.417)	(0.422)	(0.379)	(0.434)	(0.395)
Number of observations	14545	5446	4771	675	2806	2640

Source: SHS 2012, GMM II 2010, own calculations.

Notes: Reported numbers are mean values, and standard deviations are in parentheses. Sample size = 19991 observations. Only adult population up to 73 years old included. ADL is a binary variable that equals one if activities of daily living are strongly or somewhat impaired due to health problems, and zero if not impaired at all. Income refers to the equivalized monthly household income in CHF. Deductible refers to the annual health insurance deductible. The significance levels refer to the difference between the Swiss and each immigrant group: ^***^p<0.001,^**^p<0.01, ^*^p<0.05.

### Group differences in health care utilization

Table 3 summarizes the raw (not adjusted) differences in health care utilization between non-migrants and immigrants. Overall, immigrants are less likely to visit a doctor compared to non-migrants. This difference is mainly driven by first-generation and culturally different immigrants, who exhibit a significantly lower likelihood of going to the doctor. We do not find evidence for significant differences in visiting a doctor between non-migrants and second generation or culturally similar immigrants. On the other hand, all immigrant groups are more likely to visit the ED. The inequality between non-migrants and culturally different immigrants is, however, only statistically significant at the 10% level. At the intensive margin, no significant differences exist between non-migrants and immigrants in terms of the number of doctor visits. Among those visiting the ED, first-generation and culturally different immigrants exhibit a higher number of visits than non-migrants.

**Table 3:** Group differences in health care utilization

	Swiss	Immigrants				
		overall	1st generation	2nd generation	culturally different	culturally similar
Pr(doctor visit)	0.782	0.740^***^	0.736^***^	0.773	0.686^***^	0.771
		(0.450)	(0.452)	(0.439)	(0.472)	(0.418)
Pr(ED visit)	0.115	0.140^***^	0.135^**^	0.178^***^	0.135	0.143^***^
		(0.356)	(0.350)	(0.392)	(0.353)	(0.360)
Number of doctor visits if doctor visit	4.22	4.32	4.33	4.25	4.41	4.27
		(4.73)	(4.77)	(4.44)	(4.80)	(4.65)
Number of ED visits if ED visit	1.20	1.27^*^	1.28^*^	1.24	1.31^*^	1.25
		(0.52)	(0.52)	(0.56)	(0.53)	(0.51)
Number of observations	14545	5446	4771	675	2806	2640

Source: SHS 2012, GMM II 2010, own calculations.

Notes: Reported numbers are mean values, and standard deviations are in parentheses. Sample size = 19,991 observations. Only adult population up to 73 years old included. Health care utilization variables refer to past 12 months. Pr: Probability. ED: emergency department. The significance levels refer to the difference between the Swiss and each immigrant group: ^***^p<0.001,^**^p<0.01, ^*^p<0.05.

### Decomposition analysis

**Fig. 1:**
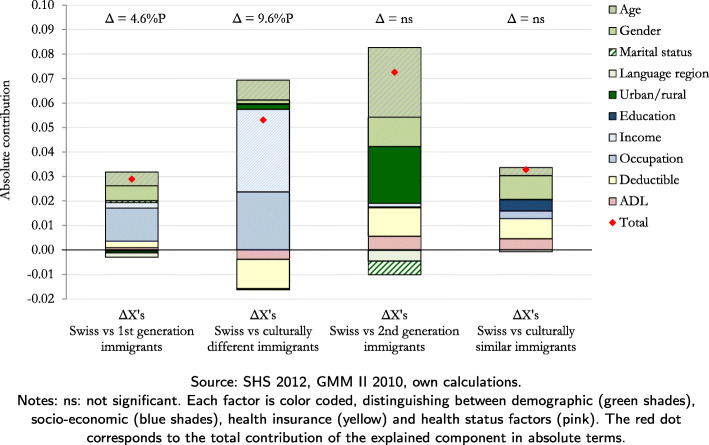
Decomposition results at extensive margin of doctor visits

**Fig. 2:**
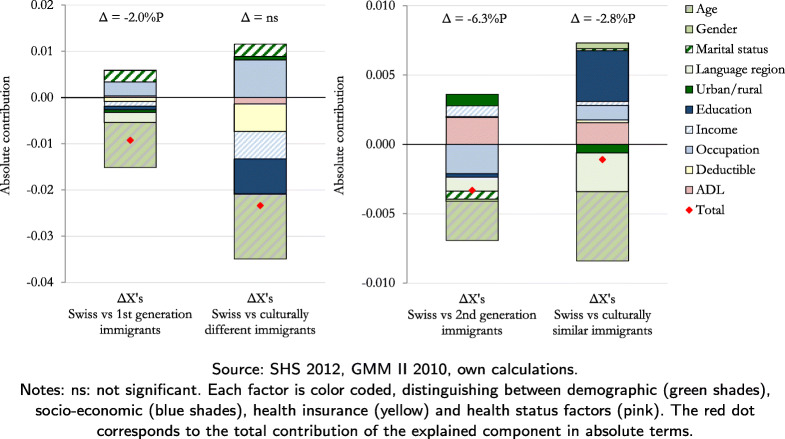
Decomposition results at extensive margin of ED visits

Table 4 and Figs. 1 and 2 present the decomposition results. Table 4 shows the contributions of the explained and the unexplained component to the inequalities at the extensive margin of doctor and ED visits. It also shows the separation of the explained component into demographic, socio-economic, health insurance and health status factors. The results are expressed in absolute coefficients, and the percentage contribution can be calculated by dividing this coefficient by the inequality (first column of Table 4). Figures 1 and 2 show the absolute contribution of each factor, color coded for demographic (green), socio-economic (blue), health insurance (yellow) and health status factors (pink).

**Table 4:** Decomposition results at extensive margin of doctor and ED visits

		Explained					Unexplained	Number of
		Demographic	Socio-economic	Health insurance	Health status	Total	Total	observations
		factors	factors	factors	factor			
**Inequality at the extensive margin of a doctor visit**								
Swiss - First- generation immigrants	= 0.046	0.010	0.015	0.003	0.001	0.029	0.017	19316
Swiss - Second-generation immigrants	= ns	0.054	0.002	0.012	0.006	0.073	-0.064	15220
Swiss - Culturally different immigrants	= 0.096	0.012	0.057	-0.012	-0.004	0.053	0.043	17351
Swiss - Culturally similar immigrants	= ns	0.012	0.008	0.008	0.005	0.033	-0.022	17185
**Inequality at the extensive margin of an ED visit**								
Swiss - First-generation immigrants	= -0.020	-0.010	0.001	-0.001	0.000	-0.009	-0.011	19316
Swiss - Second-generation immigrants	= -0.063	-0.004	-0.002	0.000	0.002	-0.003	-0.060	15220
Swiss - Culturally different immigrants	= ns	-0.011	-0.005	-0.006	-0.001	-0.023	0.004	17351
Swiss - Culturally similar immigrants	= -0.028	-0.008	0.005	0.000	0.002	-0.001	-0.027	17185

Source: SHS 2012, GMM II 2010, own calculations.

Notes: ns: not significant. The results are expressed in absolute coefficients. The observed difference shown in column two is the sum of columns seven and eight.

Our results indicate that inequalities at the extensive margin of doctor visits are attributed more to the explained (differences in characteristics) than the unexplained (differences in coefficients) component. In particular, if first-generation immigrants had the same characteristics as non-migrants, the difference in the likelihood of going to the doctor of 4.6 percentage points could be reduced by 63% (i.e. =0.029/0.046×100). Similarly, if culturally different immigrants had the same characteristics as non-migrants, then the inequality of 9.6 percentage points could be reduced by 55% (i.e =0.053/0.096×100). The leading determinants of these inequalities, for both first-generation and culturally different immigrants, are socio-economic factors. In particular, occupation and income have a combined positive contribution of 34% in the inequality with respect to first-generation immigrants, and 60% in the inequality with respect to culturally different immigrants (Fig. 1). The positive sign of these contributions indicates a reduction of the inequalities if these immigrant groups had the same occupation and income distributions as non-migrants. The level of health insurance deductible has also a relatively high contribution to the inequality at the extensive margin of doctor visits with respect to culturally different immigrants. This contribution is, however, negative (-12%) indicating that it could increase this inequality.

The inequalities at the extensive margin of ED visits are attributed more to the unexplained than the explained component. In particular, if first-generation immigrants had the same characteristics as non-migrants, the inequality at the extensive margin of ED visits of -2.0 percentage points could be reduced by 46%. For second-generation and culturally similar immigrants, the inequalities could be reduced by only 5% and 4%, respectively. The contribution of the explained component is mainly associated with differences in the demographic factors, especially in age (Fig. 2). It is also interesting to note that education has a negative contribution (positive absolute coefficient/negative inequality = negative percentage contribution) to the inequality with respect to the culturally similar immigrants, which is also relatively high (-13%). This is interesting, because the share of culturally similar immigrants that have completed a tertiary education is almost double that of non-migrants (Table 2). Therefore, given the negative contribution of education to the inequality, if the culturally similar immigrants had a lower level of education comparable to that of non-migrants, then the likelihood of them visiting the ED would increase even more. The socio-economic factors with the highest contributions are occupation and education. ADL also has a relatively high negative contribution to the inequalities with respect to second-generation (-3%) and culturally similar (-6%) immigrants.

The decomposition results regarding the insignificant differences at the extensive margin of doctor visits reveal a similar overall pattern, i.e., the relative contribution of the explained compared to the unexplained component is larger in the inequalities between non-migrants and second-generation and culturally similar immigrants. Moreover, the results indicate that the differences in the likelihood of visiting the doctor between non-migrants and the two groups of immigrants would have been even larger had they had the same characteristics. The negative difference at the extensive margin of ED visits between non-migrants and culturally different immigrants (although statistically insignificant) is mainly associated with differences in the demographic factors.

The results for the intensive margins are reported in Table S2 and Figs. S1 and S2 in the Additional Files. Due to the small differences between non-migrants and the four immigrant groups in the number of doctor and ED visits, relative contributions are difficult to interpret. However, the decomposition indicates that, if second-generation immigrants had the same characteristics as non-migrants, the number of doctor visits would have been approximately 2.9 visits higher, and the number of ED visits 0.8 higher. Regarding the other immigrant groups, the aggregate contribution of the explained component is relatively small. This is a result of partial cancelling out of factors, in particular age, education, income, occupation and ADL.

The results from the additional classification of immigrant groups are in the majority consistent with the above findings (see Tables S3, S4 and S5 and Fig. S3, S4, S5 and S6 in the Additional Files). Furthermore, immigrants from Switzerland’s neighbouring countries have a very similar decomposition as culturally similar immigrants for all outcomes. This indicates that the countries included in the culturally similar group but not included in Switzerland’s neighbouring countries, have a similar health care utilization as the latter group.

We conducted a sensitivity analysis for the statistically significant inequalities in order to assess the contribution of the need factor when self-assessed health status was used as an indicator for need instead of ADL. We found that the relative contribution of the need factor tends to increase without changing the overall pattern of the results. In some inequalities, however, the need factor changes also its sign. This might be explained by immigrants perceiving their health status differently compared to non-migrants.

### Regional sub-sample analysis

Due to the heterogeneity of non-migrants in Switzerland, in particular across language regions, we run an additional analysis of inequalities between our four immigrant groups and non-migrants by language region using the SHS 2012 data only. The results show that health care utilization is significantly higher in the French- and Italian-speaking regions compared to the German-speaking region (Table 5). The differences remain even after controlling for the predicting factors. Although the results are less precise due to the smaller sample size, they still show that health care utilization in the French- and Italian- speaking regions is rather similar, and that immigrants in these two regions follow a rather similar pattern. Thus, not distinguishing between French- and Italian-speaking regions does not come at a major loss for the inequality analysis. To this end, Figs. 1 and 2 show that the contribution of living in a German- vs. a Latin-speaking region is not highly associated with most of the health care inequalities. The association with the inequalities at the extensive margin of doctor visits is equal to -4% and 0% for first-generation and culturally different immigrants, respectively. The association with the inequalities at the extensive margin of ED visits varies between 2% and 11%.

**Table 5:** Sub-sample regression results for all outcomes using only SHS 2012 and controlling for interactions

	Pr(doctor visit)			Nr of doctor visits if doctor visit			Pr(ED visit)			Nr of ED visits if ED visit		
	Logit			NegbinII			Logit			NegbinII		
FR	0.196^***^	0.195^***^	0.195^***^	0.049^*^	0.049^*^	0.049^*^	0.236^***^	0.236^***^	0.236^***^	-0.009	-0.009	-0.009
	(0.050)	(0.050)	(0.050)	(0.022)	(0.022)	(0.022)	(0.061)	(0.061)	(0.061)	(0.024)	(0.024)	(0.024)
IT	0.252^**^	0.253^**^	0.250^**^	0.029	0.030	0.030	0.506^***^	0.506^***^	0.506^***^	0.045	0.046	0.046
	(0.090)	(0.090)	(0.090)	(0.036)	(0.036)	(0.036)	(0.095)	(0.095)	(0.095)	(0.041)	(0.041)	(0.041)
Immigrants	0.032			0.069^*^			0.204^*^			0.050		
	(0.076)			(0.035)			(0.092)			(0.041)		
FR × Immigrants	-0.265^*^			-0.114^*^			0.028			-0.034		
	(0.123)			(0.055)			(0.143)			(0.058)		
IT × Immigrants	-0.075			-0.230^*^			-0.364			-0.122		
	(0.200)			(0.095)			(0.236)			(0.076)		
1st gen IMM=1		-0.009			0.072			0.186			0.053	
		(0.080)			(0.038)			(0.100)			(0.044)	
FR ×1st gen IMM=1		-0.266^*^			-0.126^*^			0.038			-0.019	
		(0.129)			(0.059)			(0.152)			(0.062)	
IT ×1st gen IMM=1		-0.132			-0.221^*^			-0.300			-0.111	
		(0.207)			(0.103)			(0.250)			(0.079)	
2nd gen IMM=1		0.329			0.048			0.316			0.037	
		(0.206)			(0.084)			(0.213)			(0.101)	
FR ×2nd gen IMM=1		-0.177			-0.015			-0.020			-0.173	
		(0.343)			(0.150)			(0.354)			(0.112)	
IT ×2nd gen IMM=1		0.544			-0.323^*^			-1.007			-0.323^**^	
		(0.717)			(0.136)			(0.580)			(0.106)	
culturally different IMM=1			-0.011			0.099			0.258			0.058
			(0.135)			(0.063)			(0.154)			(0.061)
FR × culturally different IMM=1			-0.615^*^			-0.229^*^			-0.216			-0.016
			(0.240)			(0.113)			(0.291)			(0.098)
IT × culturally different IMM=1			-0.413			-0.306			-0.721			-0.098
			(0.389)			(0.219)			(0.480)			(0.141)
culturally similar IMM=1			0.055			0.048			0.154			0.043
			(0.080)			(0.037)			(0.097)			(0.049)
FR × culturally similar IMM=1			-0.158			-0.068			0.137			-0.034
			(0.131)			(0.059)			(0.148)			(0.067)
IT × culturally similar IMM=1			0.067			-0.184^*^			-0.148			-0.131
			(0.202)			(0.078)			(0.218)			(0.079)
Demographic factors included	Yes	Yes	Yes	Yes	Yes	Yes	Yes	Yes	Yes	Yes	Yes	Yes
Socio-economic factors included	Yes	Yes	Yes	Yes	Yes	Yes	Yes	Yes	Yes	Yes	Yes	Yes
Health insurance factors included	Yes	Yes	Yes	Yes	Yes	Yes	Yes	Yes	Yes	Yes	Yes	Yes
Health status factor included	Yes	Yes	Yes	Yes	Yes	Yes	Yes	Yes	Yes	Yes	Yes	Yes
Mean (Standard deviation)	0.78 (0.417)	0.78 (0.417)	0.78 (0.417)	4.26 (4.714)	4.26 (4.714)	4.26 (4.714)	0.12 (0.329)	0.12 (0.329)	0.12 (0.329)	1.22 (0.560)	1.22 (0.560)	1.22 (0.560)
Number of observations	17419	17419	17419	13591	13591	13591	17419	17419	17419	2096	2096	2096
Number of regressors	28	31	31	28	31	31	28	31	31	28	31	31
Pseudo R-Squared	0.098	0.099	0.099	0.040	0.040	0.040	0.037	0.037	0.038	0.005	0.005	0.005
*p*-value (F-statistic)	0.000	0.000	0.000	0.000	0.000	0.000	0.000	0.000	0.000	0.000	0.000	0.000
AMPE of IMM/FG/DIF	-0.007	-0.014	-0.034	0.092	0.094	0.079	0.019	0.018	0.015	0.035	0.045	0.054
	(0.009)	(0.010)	(0.018)	(0.117)	(0.125)	(0.220)	(0.008)	(0.008)	(0.015)	(0.036)	(0.038)	(0.059)
AMPE of SG/SIM		0.046	0.003		0.095	0.066		0.027	0.020		-0.048	0.025
		(0.022)	(0.010)		(0.290)	(0.124)		(0.020)	(0.008)		(0.081)	(0.042)

Sources: SHS 2012, own calculations.

Notes: FR: French-speaking regions. IT: Italian-speaking regions. The outcome variables refer to the past 12 months. Robust standard errors are in parentheses. The demographic factors included are age groups, gender, marital status, language region and urban region. The socio-economic factors included are education, income and occupation. The health insurance factors included are the different levels of deductible. The health status factor included is the likelihood of activities of daily living impairment (ADL).

^***^p<0.001,^**^p<0.01, ^*^p<0.05.

## Discussion

In this study, we examine the factors that are associated with inequalities in health care utilization between immigrants and non-migrants in Switzerland. We find that first-generation and culturally different immigrants have a lower likelihood of visiting the doctor, while all immigrant groups are more likely to visit the ED. By applying a non-linear decomposition, we find that inequalities at the extensive margin of doctor visits could be reduced by 63% and 55%, respectively, if the corresponding immigrant groups had the same characteristics as non-migrants. These inequalities are mainly associated with socio-economic factors and, in particular, occupation and income. On the other hand, we find that inequalities at the extensive margin of ED visits are mainly associated with the unexplained component, i.e., differences in coefficients. Hence, if the corresponding immigrant group had the same characteristics as non-migrants, the inequality at the extensive margin of ED visits between non-migrants and first-generation immigrants could be reduced by 46%, the inequality between non-migrants and second-generation immigrants by only 5% and the inequality between non-migrants and culturally similar immigrants by only 4%. Differences in the coefficients may arise from unobserved factors that affect health care utilization [31], such as health literacy, health care preferences, but also systemic barriers or discrimination. They might, however, also capture random noise.

Previous research showing worse health outcomes in some immigrant groups in Switzerland [5, 40] indicate that the healthy immigrant effect likely does not explain the inequalities in doctor visits that we observe. Our findings rather suggest that barriers might exist in the Swiss health system that prevent immigrants from using primary care, leading to a higher use of emergency services. This is observed also in other European countries [9, 41].

Such systemic barriers are at least partially reflected in the unexplained component and result from language problems, lack of health system knowledge or access restrictions. Language problems cause difficulties in communicating with health care providers, accessing and understanding health information, and eventually affect health status [42, 43]. While the SHS 2012 does not include information on language skills, according to GMM II, for 88% of the immigrants their native language differs from the one spoken in Switzerland. Notably, one third of the first-generation and culturally different immigrants were at least sometimes not able to have doctors understand their health concerns. In addition, one third at least sometimes failed to understand the information provided by the doctor. For second-generation and culturally similar immigrants these shares are considerably lower (10% and 14%). Lack of health system knowledge might also explain unequal use of health care services. According to our data, immigrants are 1.75 to 2.5 times more likely to not know their health insurance plan as well as the level of their deductible compared to non-migrants (Table 2). As our results show, not knowing the level of its own health insurance deductible explains a considerable part of the difference in health care use, especially between non-migrants and culturally different immigrants. Finally, two factors might indicate access restrictions. Firstly, most immigrant groups are more likely to not have a general practitioner (GP) compared to non-migrants (14% of the first-generation, 9% of the second-generation, 12% of the culturally different and 15% of the culturally similar immigrants compared to 9% of non-migrants). Since GPs act as the gatekeeper to specialist or hospital treatment, they help to avoid unnecessary ED visits. Secondly, although all residents are covered by universal health care, out-of-pocket payments are exceptionally high in Switzerland [44]. At the same time, most immigrant groups have a lower equivalized monthly household income, and a higher share of immigrants are unemployed compared to non-migrants (Table 2). As a result, they might be less willing or less capable to spend money for health care services.

Moreover, heterogeneous effects across immigrant groups suggest that unobserved cultural factors are related to health care utilization. Alonso et al. [45], for example, show that stigmatization and cultural background associated especially with norms towards help- and care-seeking can influence the utilization of health services.

A limitation of the decomposition used in this study is that the results cannot be given a causal interpretation [31]. Although we chose the predictors based on Andersen’s model of health services use, endogeneity might exist due to omitted variable bias. We therefore carefully interpret our results as associations, instead of causal effects. Another limitation of decomposition methods is the sensitivity of the results when the reference and comparison groups are switched, known as the indexing or identification problem [46–48]. In our case, however, this does not pose a problem per se, but is rather a matter of choosing the groups meaningfully with respect to the research question. As our aim is to evaluate how the inequality would change if immigrants were and acted the same as non-migrants, we fix the coefficients in the explained component to the level of the immigrant group.

Another limitation is that we can only account for factors that are observed in both immigrant and non-migrant groups. Hence, migration-specific characteristics that could affect health care utilization, such as language, lack of knowledge of the Swiss health care system, reasons for migrating and other pre- and post-migration factors, are not included in the decomposition analysis. While not directly included in the model, these factors might be indirectly gauged by the unexplained component of the examined inequality. We also partly accounted for this aspect in the sub-sample analysis.

To what extent are our results policy relevant? Our results offer a better understanding of the inequalities in health care utilization. This can be key for the design of interventions towards the improvement of population health and a sustainable solidarity-based health care system. Inadequate or inappropriate health care utilization of certain services may lead to poorer health, higher health inequalities, and higher healthcare expenditures [49, 50]. Ensuring, for example, equal access to primary care, equal quality of care and orienting all patients efficiently through the healthcare system could increase preventive care and reduce unnecessary hospitalizations and ED visits [51, 52].

Better health care access can be achieved with mutable factors or behavioral change [28]. While predisposing factors are less mutable, enabling factors could change with the proper interventions and could in turn benefit health care utilization. Boes and Gerfin [53], for example, show that full insurance has a significant impact on consumers’ willingness to generate costs, and in turn on their health care utilization. Need factors describing perceived and evaluated health endowment could also be influenced by improving, for example, health literacy. Eichler, Wieser and Brügger [54] show that low health literacy is associated with higher health care expenditures per person-year. This could be especially relevant for low-income groups, who are often characterized by poorer health conditions and specific health care utilization patterns [54].

It is also important to note that not only individual level factors are associated with health care inequalities. Although, for example, people with a higher level of education might have more resources to make informed and effective health care utilization choices, the systemic barriers of the country may affect this relationship [55]. These barriers refer to the financing, provision and regulation of the health care system [55, 56]. For example, recent market reforms, from universal to private, market-based health systems in many European countries, have led to restrictive health care access for immigrants [57]. This study shows that even in a country with universal health care coverage systemic barriers may exist and especially for the less integrated immigrants (i.e. first-generation and culturally different immigrants).

## Conclusion

This study assessed the inequalities in health care utilization between immigrants and non-migrants in Switzerland. The results revealed the factors most associated with these inequalities and the ways these associations take place. These results are policy relevant, as they show that migrant-specific policies should be developed that target both individual as well as institutional level changes. Through behavioral change and equal access to primary care, overall population health could be improved and health inequalities could be reduced.

## Supplementary Information

**Additional file 1** Additional tables and figures

## Data Availability

The data that support the findings of this study are available from the Swiss Federal Statistical Office and the Swiss Federal Office of Public Health but restrictions apply to the availability of these data, which were used under license for the current study, and so are not publicly available. Data are, however, available from the Swiss Federal Statistical Office and the Swiss Federal Office of Public Health upon reasonable request and under a license agreement.

## Abbreviations

EU: European union
EFTA: European free trade association
ED: Emergency department
SHS: Swiss health survey
GMM: Health monitoring of the migrant population in Switzerland (in German: Gesundheitsmonitoring der Migrationsbevölkerung)
ZEMIS: Central information system for immigrants (in German: Zentrales Migrationsinformationssystem)
OECD: Organisation for economic co-operation and development
ADL: Activities of daily living
SUI: Swiss
IMM: Immigrants
LPM: Linear probability model
BIC: Bayesian information criterion
AIC: Akaike information criterion
ns: Not significant
Pr: Probability
FR: French-speaking regions
IT: Italian-speaking regions

## Footnotes

1 We specified multivariate regression models based on Andersen’s model and first compared the predicted mean, minimum and maximum values, and then compared the Bayesian information criterion (BIC) and Akaike information criterion (AIC) with the non-linear models. Based on these comparisons, we opted for logit models for the binary outcomes and for negative binomial II for the count data outcomes.
