# Supplementary material for: What explains the inequalities in health care utilization between immigrants and non-migrants in Switzerland?
Source: BMC Public Health. 2021 Mar 18;21:530. doi: 10.1186/s12889-021-10393-9 (PMC7977586; doi:10.1186/s12889-021-10393-9)
Supplement: Supplementary file 1 — Additional file 1 Additional tables and figures [file 12889_2021_10393_MOESM1_ESM.pdf]

Additional Files

Additional Tables

Table S.1 Description of all variables

Variable	Description
**Predictors of health care utilization**	
age	Age is restricted between 17 and 73 years old and five age categories were created.
male	Binary indicator of gender equaling one if individual is male and zero if individual is female.
married	Binary indicator of civil status equaling one if individual is married and zero if individual is not married.
German-speaking region	Binary indicator of language region equaling one if region is German-speaking and zero if region is French- or Italian-speaking.
urban	Binary indicator of urban region equaling one if region is urban and zero if region is rural.
education	Categorical variable including compulsory, secondary and tertiary education.
household income	Equivalized monthly household income.
occupation	Categorical variable including the following occupation groups: intern, ordinary employee, employee with leadership, director or chief, self-employed, inactive and unemployed.
deductible	Level of deductible as defined in Switzerland: = 300, = 500, = 1000, = 1500, = 2000, = 2500 and missing, when no answer was provided. The last group was included in order to keep individuals in the sample that do not know the level of their deductible. Such individuals could, for example, be young adults who are insured together with their parents.
ADL	Binary indicator of self-assessed chronic illness that equals one if activities of daily living have strongly or somewhat been impaired due to health problems during the past six months, and zero if they have not been impaired at all.
**Health care utilization measures**	
extensive margin of doctor visits	Binary indicator showing the probability of visiting the doctor based on whether the individual self-reported as having visited a doctor in the past twelve months. This includes all types of doctor visits (e.g. to general practitioners, specialists and gynecologists) but not to dentists. Home visits are also included but not telephone consultations.
extensive margin of ED visits	Binary indicator showing the probability of visiting the emergency department based on whether the individual self-reported as having visited the emergency department in the past twelve months. This only refers to unplanned visits.
intensive margin of doctor visits	Count indicator of the self-reported number of doctor visits, if the individual had at least one visit during the past twelve months.
intensive margin of ED visits	Count indicator of the self-reported number of emergency department visits, if the individual had at least one visit during the past twelve months.

Table S.2 Decomposition results at intensive margin of doctor and ED visits

		Explained					Unexplained	Number of
		Demographic factors	Socio-economic factors	Health insurance factors	Health status factor	Total	Total	observations
**Inequality at the intensive margin of doctor visits**								
Swiss - First-generation immigrants	= ns	0.000	-0.073	-0.004	-0.016	-0.093	-0.010	14782
Swiss - Second-generation immigrants	= ns	1.848	0.467	0.058	0.499	2.872	-2.902	11872
Swiss -Culturally different immigrants	= ns	0.152	-0.220	-0.133	-0.122	-0.323	0.152	13239
Swiss -Culturally similar immigrants	= ns	-0.039	0.055	0.067	0.109	0.192	-0.251	13415
**Inequality at the intensive margin of ED visits**								
Swiss - First-generation immigrants	= -0.1	-0.035	-0.027	0.001	0.002	-0.059	-0.021	2359
Swiss - Second-generation immigrants	= ns	0.598	0.243	-0.076	0.027	0.791	-0.845	1803
Swiss -Culturally different immigrants	= -0.1	-0.072	-0.033	-0.067	-0.002	-0.173	0.059	2084
Swiss -Culturally similar immigrants	= ns	-0.006	-0.001	0.002	0.003	-0.001	0.054	2078

Source: SHS 2012, GMM II 2010, own calculations.

Notes: ns: not significant. The results are expressed in absolute coefficients.

Table S.3 Group differences in health care utilization for different immigrant groups

	Swiss	Immigrants				
		Neighbouring countries	Other European countries	Non-European countries	Residence duration $\geq$ 10 years	Residence duration $<$ 10 years
Pr(doctor visit)	0.782	0.790	0.682***	0.766	0.757	0.711***
		(0.407)	(0.474)	(0.439)	(0.442)	(0.462)
Pr(ED visit)	0.115	0.139**	0.136**	0.153	0.133*	0.144**
		(0.347)	(0.347)	(0.383)	(0.349)	(0.360)
Number of doctor visits if doctor visit	4.22	4.41	4.21	4.35	4.65**	3.93*
		(5.00)	(4.83)	(5.43)	(5.26)	(4.80)
Number of ED visits if ED visit	1.20	1.21	1.31**	1.31	1.28*	1.27
		(0.57)	(0.68)	(0.66)	(0.65)	(0.64)
Number of observations	14545	1600	2530	1316	2635	2460

Source: SHS 2012, GMM II 2010, own calculations.

Notes: Switzerland's neighbouring countries: Austria, France, Germany, Italy, Lichtenstein. Other European countries excluding Russia and Turkey and including English-speaking OECD countries: Albania, Australia, Belgium, Bulgaria, Canada, Czech Republic, Denmark, Finland, former Yugoslavia, Greece, Hungary, Ireland, Latvia, Lithuania, Luxembourg, Malta, Moldova, Netherlands, Poland, Portugal, Romania, Slovakia, Spain, Sweden, Ukraine, United Kingdom, United States. Non-European countries: Afghanistan, Algeria, Angola, Argentina, Armenia, Bolivia, Brazil, Burundi, Cambodia, Cameroon, Cape Verde, Central Africa, Chile, China, Colombia, Congo, Costa Rica, Côte d'Ivoire, Cuba, Dominican Republic, Ecuador, Egypt, Eritrea, Ethiopia, Gambia, Georgia, Guinea, Haiti, India, Indonesia, Iran, Iraq, Israel, Japan, Kenya, Lebanon, Madagascar, Mali, Mauritius, Mexico, Mongolia, Morocco, Niger, Pakistan, Palestine, Peru, Philippines, Russia, Rwanda, Senegal, Somalia, South Africa, Sri Lanka, Thailand, Togo, Tunisia, Turkey, Uzbekistan, Venezuela.

Reported numbers are mean values, and standard deviations are in parentheses. Sample size = 19991 observations. Only adult population up to 73 years old included. Health care utilization variables refer to past 12 months. Pr: Probability. ED: emergency department. The significance levels refer to the difference between the Swiss and each immigrant group: $^{***}p<0.001$, $^{**}p<0.01$, $^{*}p<0.05$.

Table S.4 Decomposition results at extensive margin of doctor and ED visits for different immigrant groups

		Explained					Unexplained	Number of
		Demographic factors	Socio-economic factors	Health insurance factors	Health status factor	Total	Total	observations
**Inequality at the extensive margin of doctor visits**								
Swiss - Immigrants from neighbouring countries	= ns	0.012	0.003	0.006	0.003	0.024	-0.032	16145
Swiss - Immigrants from other European countries	= 0.100	-0.005	0.032	0.002	0.006	0.035	0.065	17075
Swiss - Immigrants from non-European countries	= ns	0.012	0.027	-0.009	-0.008	0.023	-0.007	15861
Swiss - Immigrants with residence duration $\geq$ 10 years	= ns	0.003	0.017	-0.005	-0.006	0.008	0.017	17180
Swiss - Immigrants with residence duration $<$ 10 years	= 0.071	0.003	0.013	0.014	0.011	0.041	0.030	17005
**Inequality at the extensive margin of ED visits**								
Swiss - Immigrants from neighbouring countries	= -0.024	-0.004	0.015	-0.001	0.002	0.012	-0.036	16145
Swiss - Immigrants from other European countries	= -0.021	-0.035	0.003	0.001	0.003	-0.028	0.007	17075
Swiss - Immigrants from non-European countries	= ns	-0.017	-0.021	-0.010	-0.003	-0.051	0.014	15861
Swiss - Immigrants with residence duration $\geq$ 10 years	= -0.018	0.001	0.000	-0.002	-0.001	-0.003	-0.015	17180
Swiss - Immigrants with residence duration $<$ 10 years	= -0.029	-0.033	-0.002	0.001	0.002	-0.031	0.003	17005

Source: SHS 2012, GMM II 2010, own calculations.

Notes: ns: not significant. The results are expressed in absolute coefficients. The observed difference shown in column two is the sum of columns seven and eight.

Table S.5 Decomposition results at intensive margin of doctor and ED visits for different immigrant groups

		Explained					Unexplained	Number of
		Demographic factors	Socio-economic factors	Health insurance factors	Health status factor	Total	Total	observations
**Inequality at the intensive margin of doctor visits**								
Swiss - Immigrants from neighbouring countries	= ns	0.030	0.010	0.074	0.074	0.188	-0.369	12637
Swiss - Immigrants from other European countries	= ns	-0.054	0.033	-0.006	0.017	-0.010	0.020	13043
Swiss - Immigrants from non-European countries	= ns	0.443	-0.295	-0.170	-0.139	-0.161	0.023	12346
Swiss - Immigrants with residence duration $\geq$ 10 years	= -0.4	-0.017	-0.086	-0.134	-0.158	-0.395	-0.011	13305
Swiss - Immigrants with residence duration $<$ 10 years	= 0.3	0.107	-0.087	0.099	0.150	0.270	0.011	13070
**Inequality at the intensive margin of ED visits**								
Swiss - Immigrants from neighbouring countries	= ns	-0.003	-0.004	0.013	0.002	0.008	-0.020	1899
Swiss - Immigrants from other European countries	= -0.1	-0.138	0.016	-0.018	-0.001	-0.140	0.022	2028
Swiss - Immigrants from non-European countries	= ns	-0.065	0.021	-0.124	0.001	-0.168	0.053	1910
Swiss - Immigrants with residence duration $\geq$ 10 years	= -0.1	-0.012	-0.061	0.010	-0.005	-0.068	-0.020	2050
Swiss - Immigrants with residence duration $<$ 10 years	= ns	-0.096	-0.016	0.007	0.010	-0.094	0.022	2050

Source: SHS 2012, GMM II 2010, own calculations.

Notes: ns: not significant. The results are expressed in absolute coefficients. The observed difference shown in column two is the sum of columns seven and eight.

Additional Figures

Figure S.1 Decomposition results at intensive margin of doctor visits

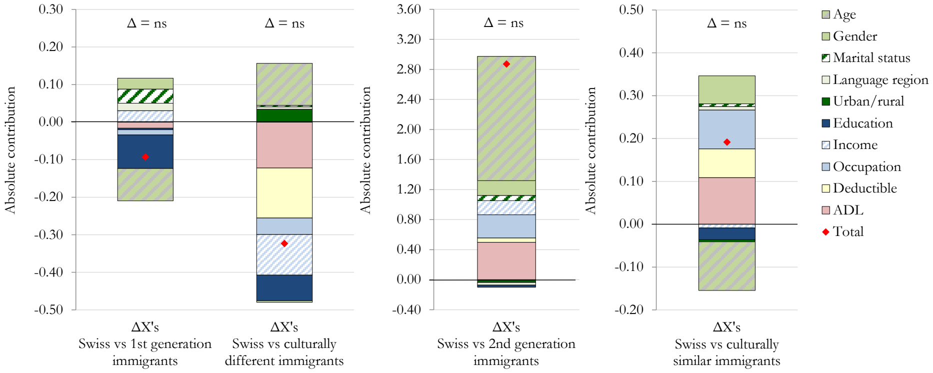

Source: SHS 2012, GMM II 2010, own calculations.
Notes: ns: not significant. Each factor is color coded, distinguishing between demographic (green shades), socio-economic (blue shades), health insurance (yellow) and health status factors (pink). The red dot corresponds to the total contribution of the explained component in absolute terms.

Figure S.2 Decomposition results at intensive margin of ED visits

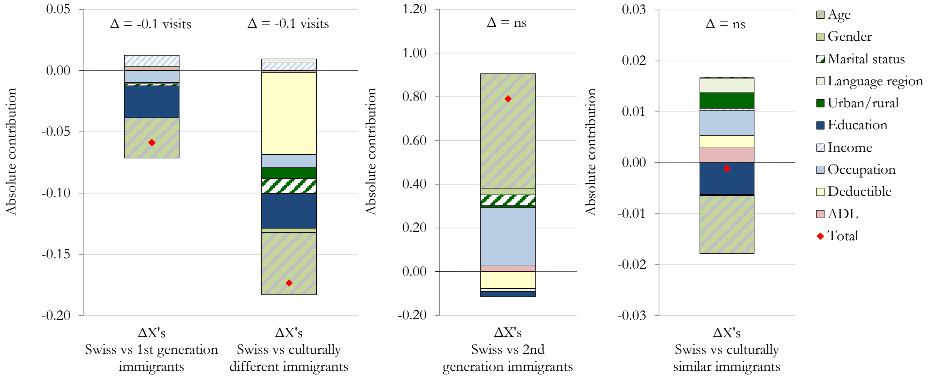

Source: SHS 2012, GMM II 2010, own calculations.
Notes: ns: not significant. Each factor is color coded, distinguishing between demographic (green shades), socio-economic (blue shades), health insurance (yellow) and health status factors (pink). The red dot corresponds to the total contribution of the explained component in absolute terms.

Figure S.3 Decomposition results at extensive margin of doctor visits for different immigrant groups

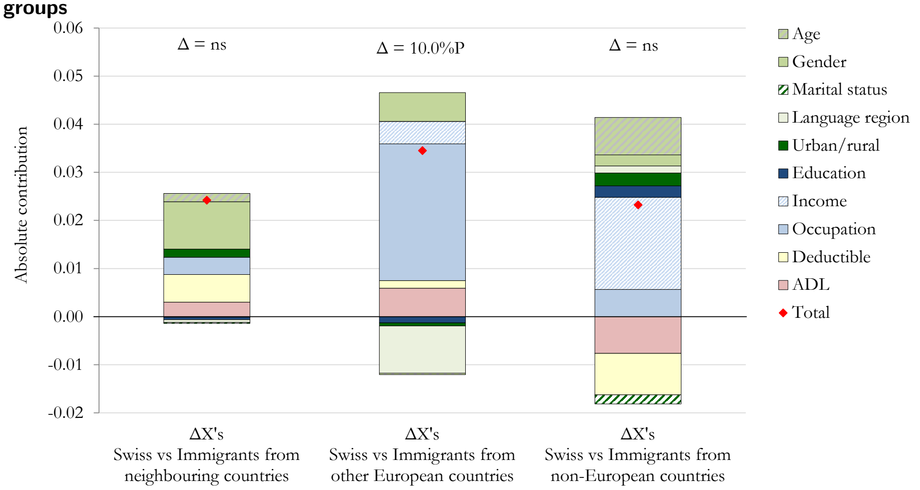

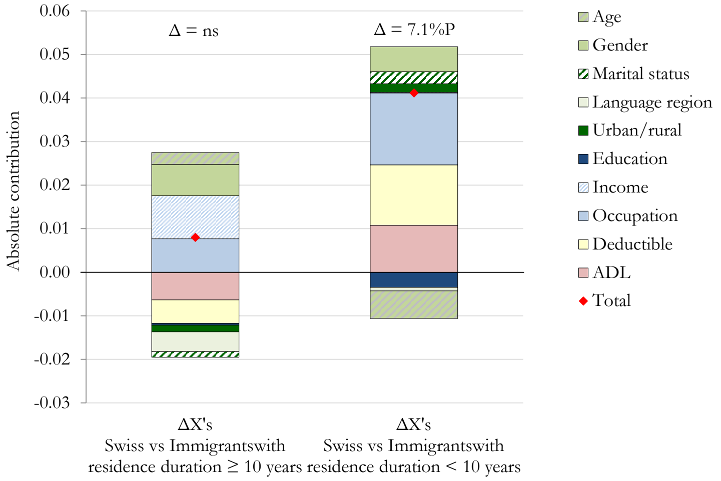

Source: SHS 2012, GMM II 2010, own calculations.
Notes: ns: not significant. Each factor is color coded, distinguishing between demographic (green shades), socio-economic (blue shades), health insurance (yellow) and health status factors (pink). The red dot corresponds to the total contribution of the explained component in absolute terms.

Figure S.4 Decomposition results at extensive margin of ED visits for different immigrant groups

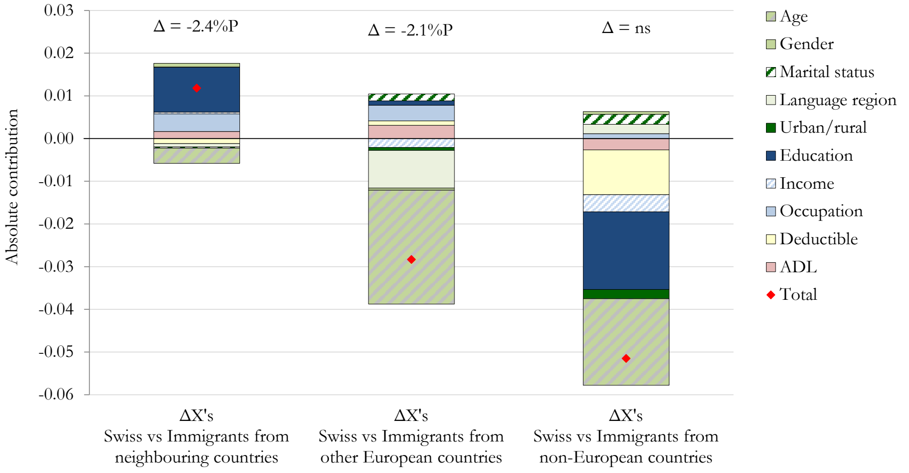

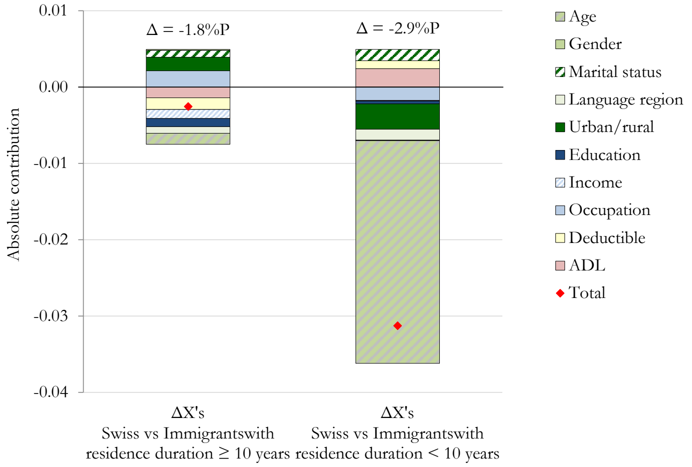

Source: SHS 2012, GMM II 2010, own calculations.
Notes: ns: not significant. Each factor is color coded, distinguishing between demographic (green shades), socio-economic (blue shades), health insurance (yellow) and health status factors (pink). The red dot corresponds to the total contribution of the explained component in absolute terms.

Figure S.5 Decomposition results at intensive margin of doctor visits for different immigrant groups

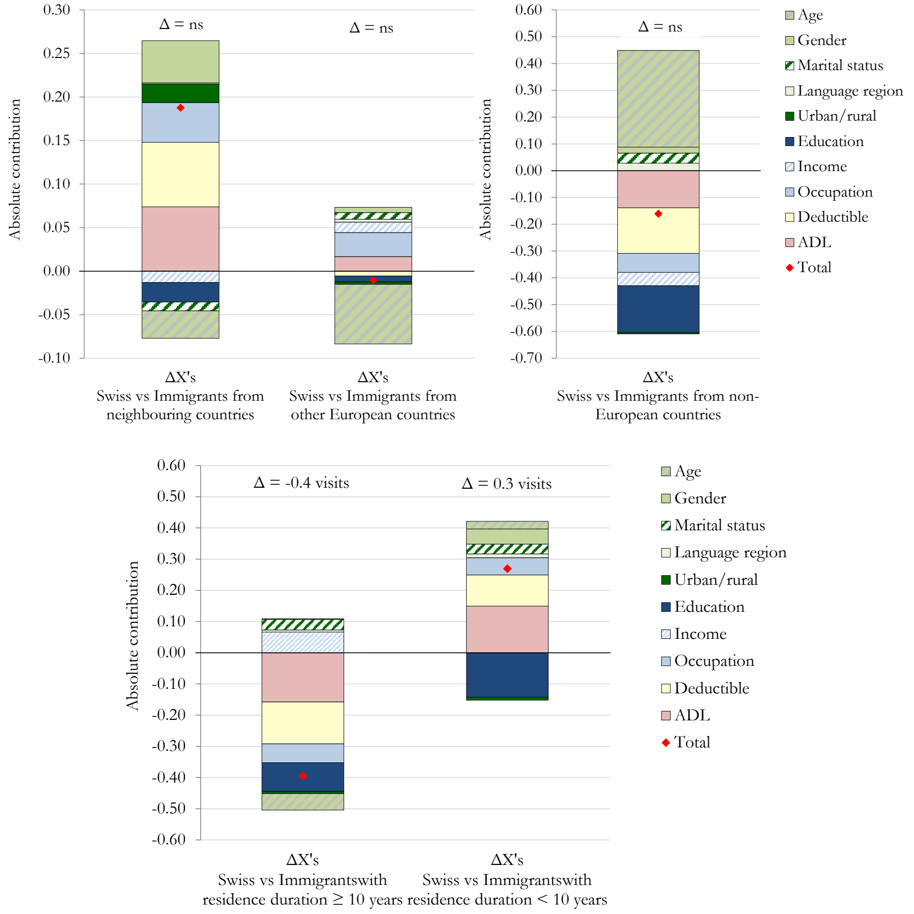

Source: SHS 2012, GMM II 2010, own calculations.
Notes: ns: not significant. Each factor is color coded, distinguishing between demographic (green shades), socio-economic (blue shades), health insurance (yellow) and health status factors (pink). The red dot corresponds to the total contribution of the explained component in absolute terms.

Figure S.6 Decomposition results at intensive margin of ED visits for different immigrant groups

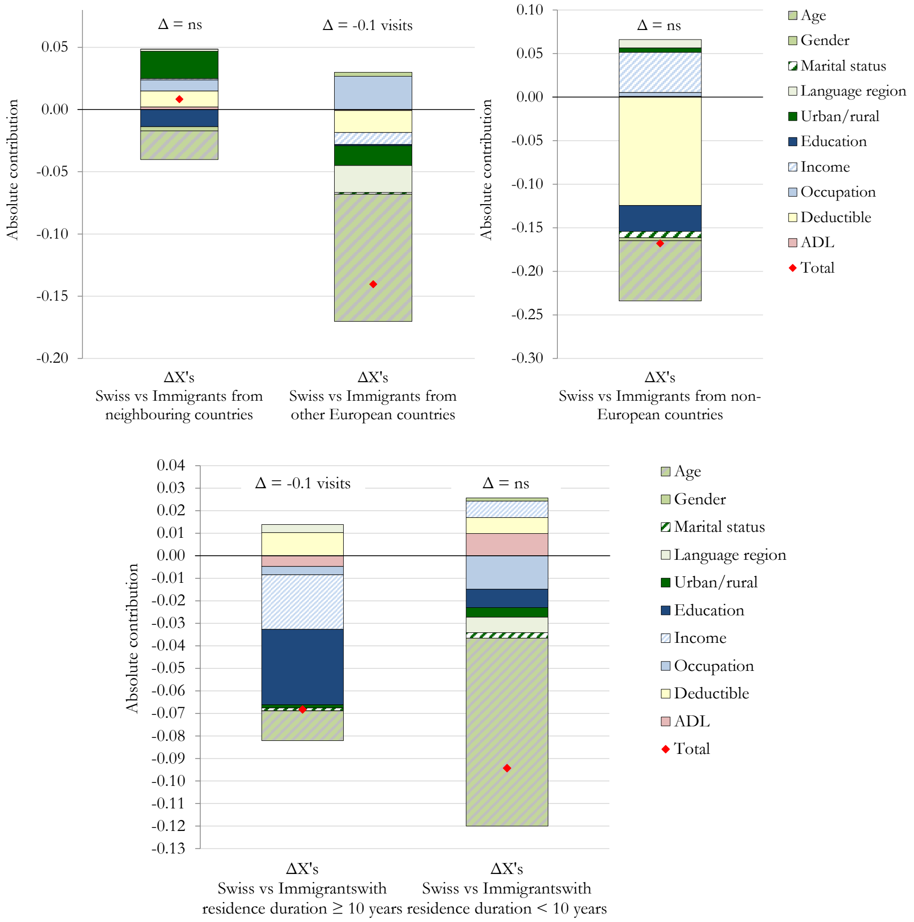

Source: SHS 2012, GMM II 2010, own calculations.
Notes: ns: not significant. Each factor is color coded, distinguishing between demographic (green shades), socio-economic (blue shades), health insurance (yellow) and health status factors (pink). The red dot corresponds to the total contribution of the explained component in absolute terms.
